# Deep learning to predict the lab-of-origin of engineered DNA

**DOI:** 10.1038/s41467-018-05378-z

**Published:** 2018-08-07

**Authors:** Alec A. K. Nielsen, Christopher A. Voigt

**Affiliations:** Synthetic Biology Center, Department of Biological Engineering, Massachusetts Institute of Technology, Cambridge, MA 02139 USA

## Abstract

Genetic engineering projects are rapidly growing in scale and complexity, driven by new tools to design and construct DNA. There is increasing concern that widened access to these technologies could lead to attempts to construct cells for malicious intent, illegal drug production, or to steal intellectual property. Determining the origin of a DNA sequence is difficult and time-consuming. Here deep learning is applied to predict the lab-of-origin of a DNA sequence. A convolutional neural network was trained on the Addgene plasmid dataset that contained 42,364 engineered DNA sequences from 2230 labs as of February 2016. The network correctly identifies the source lab 48% of the time and 70% it appears in the top 10 predicted labs. Often, there is not a single “smoking gun” that affiliates a DNA sequence with a lab. Rather, it is a combination of design choices that are individually common but collectively reveal the designer.

## Abstract (web summary)

The synthetic biology era has seen a rapidly growing number of engineered DNA sequences. Here, the authors develop a deep learning method to predict the lab-of-origin of a DNA sequence based on hidden design signatures.

## Introduction

Ted Kaczynski—the “Unabomber”—was the target of one of the longest and most expensive investigations by the FBI. He was caught when he published his 35,000-word manifesto because of similarities with earlier letters sent to his brother and newspapers: odd capitalization, linguistic idiosyncrasies, hyphen usage, and misspellings^1^. Individually, these features are not unique, but collectively they linked the documents. Similarly, his mail bombs shared design choices: 4 9 V batteries without outer casing, screws embedded in a wooden box, et cetera^2^. Again, alone these are common and widely available components, but together they pointed to a common designer.

There have been two confirmed attacks with biological weapons within the United States: the poisoning of salad bars with Salmonella in 1984 and the Anthrax letters sent in 2001. Neither involved deliberate engineering of the strain. After an insider informant pointed to the Rajneeshee cult, the subsequent attribution of the Salmonella strain relied on classical epidemiology (antibiotic susceptibility, plasmid profile, and metabolic characteristics) and a match was made to a culture tube in a clandestine lab^3,4^. Strains isolated from the Anthrax letters showed morphological features that were traced to genome mutations^5^ that aided the confirmation that the *Bacillus* *anthracis* Ames mutant was from a flask at the United States Army Medical Research Institute of Infectious Diseases at Fort Detrick^6–8^. Both cases involved extensive screening of large collections of natural isolates to search for strains with similar markers.

The synthetic biology era has seen rapid advances in the tools to engineer cells, leading to the forward design of large genetic systems^9–11^. Building a cell to make a product, such as a material or pharmaceutical, requires the introduction of dozens of genes and genetic parts and massive changes to the genome^12^. There are many design choices that leave signatures in the DNA. For example, a genetic part (e.g., a promoter) might be selected from a favorite library or because of previous positive experience. Computational design tools are increasingly prevalent^13–23^ and multiple options lead to subtle differences, for example, particular codon pairs that result from different gene optimization algorithms^24–26^. Further, DNA construction methods leave identifiable “scar” sequences^27–30^. Design choices can also lead to unintended markers, such as lab-specific mutants of plasmids or strains. Similarly, many commonly used genes, such as *lacI* or *gfp*, are reported identically in the literature but actually contain silent mutations that propagate across projects. Collectively, these design choices lead to a “signature” affiliated with an individual, lab, or institute. Even an expert eye using existing bioinformatics tools^31,32^ would find it difficult or impossible to identify signatures within a long string of nucleotides (nts), such that it could be compared against large sequence databases looking for matches for attribution.

**Fig. 1:**
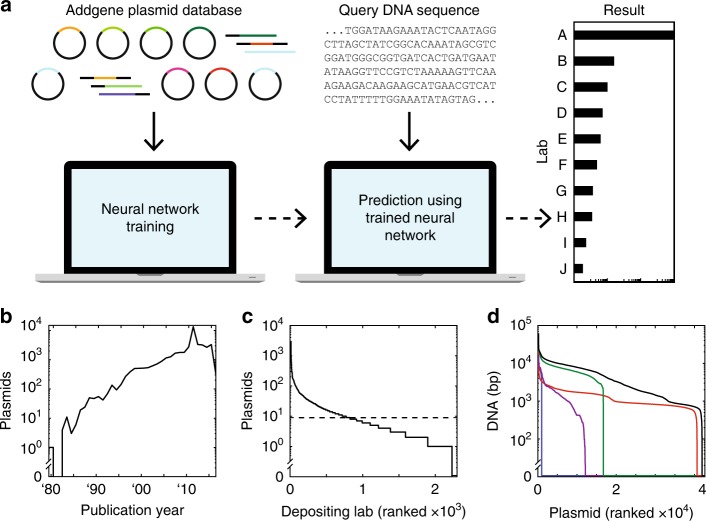
Plasmid dataset and machine learning approach. **a** Machine learning plasmid attribution strategy. **b** Plasmid publication dates across the dataset. **c** Depositing labs ranked by how many plasmids they have submitted in the dataset. A minimum cutoff of nine plasmids was enforced for training (dashed line). **d** Plasmids ranked by how much associated DNA sequence (in bp) has been submitted. DNA sequence information is categorized as Partial Depositor (purple), Full Depositor (green), Partial Repository (red), and Full Repository (blue). The summed DNA sequence length across all four categories is also shown (black). Plasmid order not maintained between the five curves

Deep convolutional neural networks (CNNs) have revolutionized image classification problems^33^. Layers of neurons are trained to identify signatures that parse images into categories, for example, to determine the identity of a face in a photograph from a set of possibilities^34^. CNNs can be trained to play checkers, Go^35^, and Atari games^36^ by processing the image of the board and selecting the next move from a set. CNNs have also been applied to categorize text based on characters, without requiring any pre-coded knowledge of words or semantic structure (e.g., news articles into “sports” and “finance”)^37^. DNA sequences have been similarly analyzed by CNNs to identify promoters^38^, regulator binding sites^37^, and other features^40–45^ by training on functional and non-functional sequences. Here we apply a similar approach to train a CNN to categorize DNA sequences to predict the lab-of-origin (“Church,” “Keasling,” “Silver”, et cetera) (Fig. 1a). This approach does not require any hand-selection of features or sequence-function information, such as part boundaries, operators, or gene annotation, which is often missing or inaccurate in public DNA sequence datasets.

## Results

### Addgene dataset

The sequences of engineered DNA are maintained in a number of large public and private repositories^46–48^, internal databases (labs, institutes, companies, consortia), appear in published patents and papers, and in the records of DNA synthesis companies. The DNA sequences are linked with other metadata, including the submitting lab. We obtained a plasmid dataset from Addgene, a nonprofit repository that archives, maintains, and distributes plasmids to the academic community^49^. Globally, labs submit their plasmids for storage and redistribution (Fig. 1b–d). It contained 42,364 plasmids (197 million nts of associated DNA sequence) deposited by 2,230 labs (Methods).

Prior to analysis, we pre-processed the DNA sequence data and removed labs with fewer than nine deposited plasmids (Methods). This resulted in a set of 36,764 plasmid sequences from 827 labs. These were then divided into three groups. To ensure representation from all labs, three plasmids from each lab were selected randomly for validation and an additional three for cross-validation. The training set comprises the remaining 31,802 sequences.

### Neural network architecture and performance

There are many ways to design a neural network. Initially, we tried different approaches including alternative architectures for a CNN (Supplementary Figure 1 and Supplementary Note 1A) as well as a recurrent neural network including long short-term memory units between the convolutional and fully connected layers (Supplementary Figure 2 and Supplementary Note 1B)^43^. A description and comparison of these methods is provided in Supplementary Information. After evaluating these approaches, we selected a CNN architecture (described below) that provided the best performance, is straightforward to implement, and relatively simple to interpret.

**Fig. 2:**
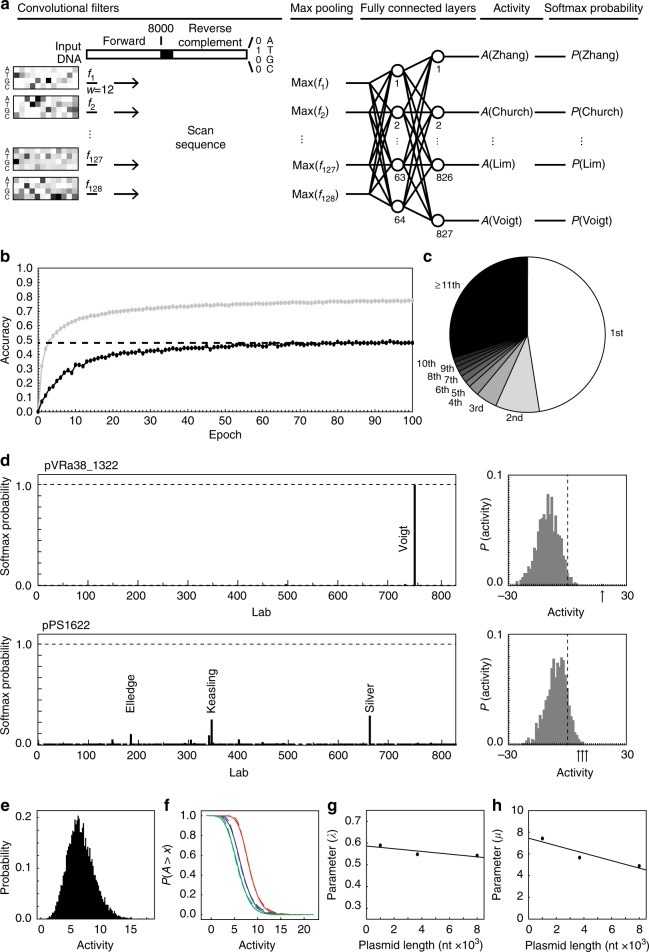
Convolutional neural network accuracy. **a** Convolutional neural network (CNN) architecture. DNA sequences are converted to 16,048 × 4 matrices, where the identity of each nucleotide is converted to a one-hot vector. This input is scanned by 128 convolutional filters (*f*_*1*_–*f*_*128*_) each with a width, *w*, of 12 nucleotide positions. Per-position nucleotide filter weights are converted to Boltzmann factors and visualized using a heatmap (Methods). The maximum activation for each filter, max*(f*_*k*_*)*, across the entire input sequence is taken. Activations are fed through two fully connected layers, which generates neural activity predictions for each lab, *A*(Name), before behind converted to probabilities using the softmax function, *P*(Name). The lab prediction is taken as the highest softmax probability. Batch normalization layers are not shown. **b** Training accuracy (gray) and validation accuracy (black) per epoch for the chosen architecture. Cross-validation accuracy was computed after training (dashed line). **c** Output prediction rank of the actual lab-of-origin for plasmids in the cross-validation set. **d** Neural network softmax probabilities (left panel) for a Christopher Voigt lab plasmid (*pVRa38_1322*) and a Pamela Silver lab plasmid (*pPS1622*). Labs with the highest probabilities are labeled. Normalized distribution of pre-softmax neuron activity (“Activity”, right panel) for the plasmids of interest. Arrows highlight the activity for labeled labs at left. The vertical dashed lines mark the origin. **e** Normalized distribution of activity for 10^4^ random DNA sequences with length 3685 nt. **f** *P* value distributions for random DNA sequences for lengths 8000, 3685, and 1000 nt (from left to right). Empirical data (solid lines) and fits to *P*(*A* > *x*) = 1−exp(−exp(−*λ*(*x*−*μ*))) (dashed lines) are shown. **g** Distribution fit steepness parameter (*λ*) versus plasmid length with a trend of *λ* = 0.59–6.2 × 10^−6^*x* (dashed line). **h** Distribution fit offset parameter (*μ*) versus plasmid length with a trend of *μ* = 7.5–3.4 × 10^−4^*x* (dashed line)

The input to the CNN is the DNA sequence encoded as a 16,048 × 4 matrix, where the identity of each nt is represented by a one-hot vector (Fig. 2a). All sequences are 8000 nts (shorter sequences are extended with Ns and longer sequences are truncated) and the reverse complement is also included, separated by 48 Ns to avoid positional effects between the two. This feeds into a single convolutional layer of 128 filters, each effectively a sliding window of 12 nts. The number of convolutional layers, number of filters, and window size were determined via Bayesian optimization (Methods, Supplementary Figure 3, and Supplementary Note 1C). A max-pooling layer is applied to each filter, which reduces the size of the representation. It also eliminates the position dependence of features along the DNA sequence. The max-pooled signals from the 128 filters then feed into two fully connected layers of 64 and 827 nodes, the latter of which corresponds to the number of labs. The second fully connected layer generates outputs for each lab, which are converted to probabilities using the softmax function (Methods). These probabilities represent the relative strength of the prediction that a query DNA sequence is associated with each category (lab) and are normalized to sum to unity across categories.

This architecture consists of 1019 neurons and 69,051 parameters, which is relatively small compared to image-processing CNNs^33,50,51^. Parameterization was implemented over 100 epochs, after which a training accuracy of 77% was achieved (with a 48% validation accuracy and 48% cross-validation accuracy) (Fig. 2b). Training required 21 h using a single NVIDIA GRID K520 GPU. After training, the filters are conceptually similar to 12 nt position weight matrices, a form often used in genetics for motif detection and reporting. The 128 filters are weighted differently across labs. Once trained, the CNN is able to rapidly analyze a sequence data stream at 980,000 bp s^−1^ on the same GPU (Methods). This is sufficiently fast for a single processor to continuously scan global DNA synthesis orders (32 bp s^−1^, 2015^52^) and sequence deposits to the largest genomics database (667 bp s^−1^, Genbank 2016^46^).

On cross-validation sequences not used for training or validation, the CNN is able to predict the correct lab-of-origin 48% of the time, as ranked by activity (Fig. 2b). Random selection of a depositing lab out of the 827 would correspond to an accuracy of 0.12%. Further, 70% of the time, the correct lab is ranked within the top 10 predicted labs (Fig. 2c). For a new query sequence, an activity is calculated for each lab in the set. Two examples are shown in Fig. 2d: one has a strong prediction where the activity of the correct lab is far from the distribution and the other where the correct lab is identified, but it is much closer to the distribution. This is captured by the softmax probability, where a higher probability corresponds to a prediction that is further from the activities of other labs. Simulations are used as a means of determining the statistical significance of sequence comparisons^53,54^. This allows for the calculation of the likelihood that a match is random given the sequence length and size of the database. We applied this approach to calculate *P* values associated with activities (Methods and Fig. 2e–h). The *P* values for the two examples in Fig. 2d are 0.0011 and 0.65, respectively. This quantifies the significance of the first prediction, despite both having approximately the same activities.

### Comparison with BLAST

The most common tool for comparing DNA sequences is BLAST (Basic Local Alignment Search Tool), designed to determine whether sequences are evolutionarily related^31^. BLAST seeks to identify target sequences in a database that share long stretches of identical nts with the query sequence. For our purposes, this is not ideal because many plasmids across labs share long stretches of sequences due to similar backbones, antibiotic resistances, fluorescent reporters, and so on. Searches against large databases are swamped by hits that share the long features. In contrast, the CNN focuses on short unique regions that maximally aid classification.

**Fig. 3:**
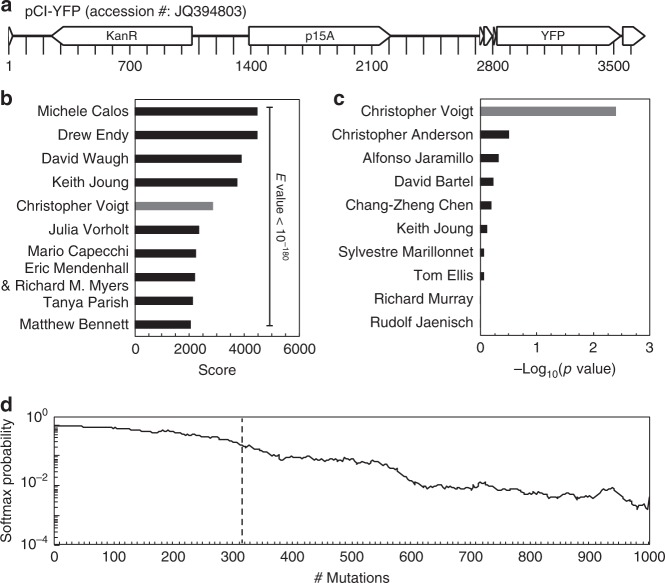
Statistical analysis of neural network and BLAST results. **a** Plasmid map for *pCI-YFP* with labeled major genetic features shown. **b** BLAST score for *pCI-YFP* alignments to plasmid training set (depositing lab shown instead of plasmid name). **c** *P* values for *pCI-YFP* lab predictions calculated from activities. **d** Effect of point mutations on the Voigt lab softmax probability for *pCI-YFP*. The geometric mean of 30 trajectories of random mutations is shown, with the average number of mutations that cause an incorrect top prediction (dashed line)

When BLAST is used to compare sequences with the Addgene dataset, it identifies the correct lab with 70% accuracy, slightly lower than the CNN training accuracy. The overfitting is apparent when it is used to evaluate sequences outside of training or validation, for example, from Genbank. This effect is illustrated by an example in Fig. 3 using a plasmid from the Voigt lab (*pCI-YFP*, JQ394803.1) that is present in Genbank but not in the Addgene dataset. This represents a hard plasmid for attribution using database information because 64% of its 3685 bp are made up of extremely common sequences (a *p15A* origin of replication, *kanR*, and *yfp*). There are many matches for these sequences (*E*-value < 10) in both the Addgene dataset/Genbank databases: 562/13904, 502/3620, and 692/1668, respectively. As such, BLAST is unable to identify the correct lab from the plasmid training dataset, from which 11,369 plasmids have an *E*-value < 10^−180^ and the closest Voigt lab plasmid ranking 5th (Fig. 3b). In contrast, the CNN correctly predicts *pCI-YFP* as being from the Voigt lab by a large difference (Fig. 3c). Further, using BLAST, we identified the next 16,000 matches from Genbank that align to *pCI-YFP*, all of which have very low *E*-values. When these are analyzed using the CNN, only 3% are assigned to the Voigt lab out of the 827 options (a random assignment corresponds to 0.1%).

### Sensitivity to mutations

The impact of DNA sequence point mutations on the predictions was then assessed. Computationally, random mutations were made to the *pCI-YFP* plasmid and the CNN prediction re-evaluated. These mutations could represent sequencing errors, genetic drift, or deliberate directed evolution. On average, 316 mutations could be made to the sequence (changing ~9% of the plasmid) before the correct prediction was lost (Fig. 3d). This demonstrates that the lab-specific signature is robust and would be difficult to erase via a scheme of laboratory-driven neutral evolution^55–57^.

### Interpretation of predictions

It is useful to identify the regions of a DNA sequence that correspond to its assignment to a particular lab. Note that this is not always possible, as it is notoriously difficult to interpret how neural networks make predictions^58^. To this end, we developed a method to visualize regions that contribute strongly to the CNN prediction. Following an approach described by Solovyev and Umarov^39^, a window of 50 Ns is scanned across the plasmid to obscure the underlying DNA sequence (Methods). The activities for the labs are calculated for each window position. A drop in the activity for a lab indicates that that sequence contributes to the prediction and if the activity goes up, it can be interpreted that this region makes it look less like it came from the lab. Note that this analysis only shows the first-order connections between sequence and the prediction and will not reveal higher-order effects requiring multiple regions (see below).

**Fig. 4:**
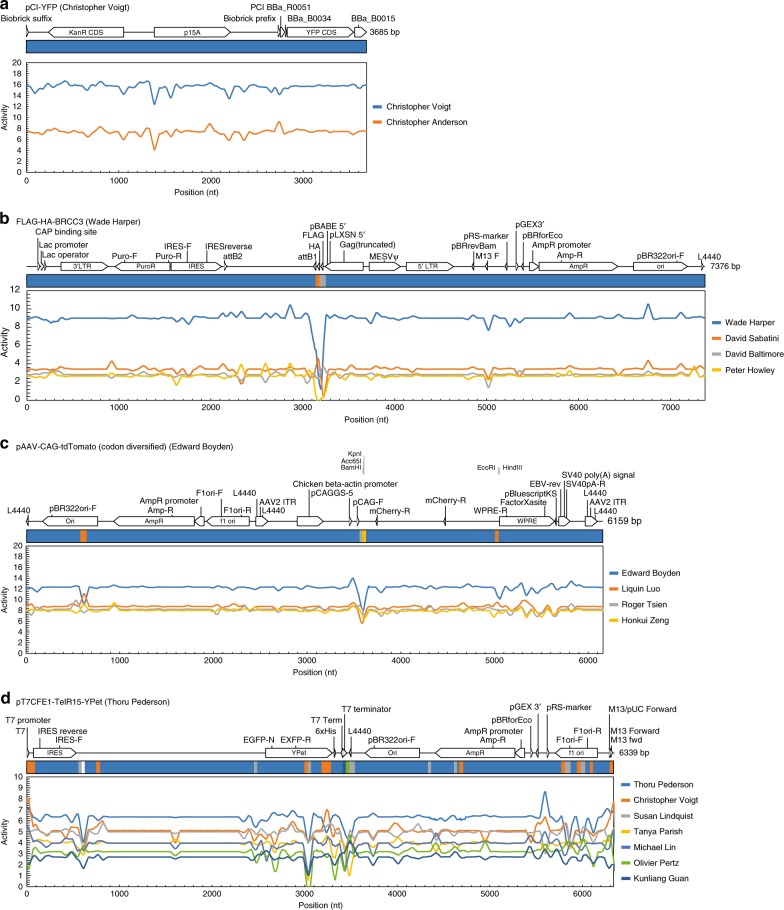
Plasmid disruption analysis. Sequence scanning for plasmids **a** *pCI-YFP* from Christopher Voigt’s lab, **b** *FLAG-HA-BRCC3* from Wade Harper’s lab, **c** *pAAV-CAG-tdTomato* *(codon diversified)* from Edward Boyden’s lab, and **d** *pT7CFE1-TelR15-YPet* from Thoru Pederson’s lab. Genetic schematics with feature annotations from the plasmid dataset file shown at the top of each panel. Colored bars display the lab prediction when the corresponding region of the plasmid is obscured by a sliding window of 50 Ns (widths not to scale); colors correspond to the lab legend (right) and white corresponds to a lab not present in the legend. Colored traces show the average pre-softmax neural activity (“Activity”) for top predicted labs as each region of the plasmid is obscured. For each nucleotide position, average activations are calculated for all 50 frames where the disruption window obscures that position

Profiles were generated for all of the plasmids tested during cross-validation. Several classes of effects were observed and representative examples are shown in Fig. 4. For most plasmids, there is no simple combination of genetic signals to guide classification, and deep learning is needed to learn complex mappings from DNA sequence. One example is the *pCI-YFP* plasmid, whose profile is relatively flat for the entirety of the sequence and obscuring no 50 nt region causes it to be improperly classified (Fig. 4a).

In some cases, a “smoking gun” sequence could be identified that was strongly associated with a lab and the prediction changes drastically when this sequence is obscured. This can be from a deliberate design objective, a secondary consequence (e.g., DNA construction methodology or at part junctions), or accidentally due to a known or unknown silent mutation associated with a part (e.g., a synonymous mutation in a fluorescent reporter). An example of a deliberate design objective is the *FLAG-HA-BRCC3* plasmid submitted by Wade Harper’s lab (Fig. 4b). This plasmid contains a *FLAG-HA* affinity tag, whose presence is central to the design of the plasmid (and the “*FLAG-HA*” in the plasmid name). When this region of the plasmid is obscured, the plasmid can be made to look like two other labs (David Sabatini’s and David Baltimore’s), depending on the position of the masking window. This is due to the prevalence of other plasmid features (*pBR322* origin of replication, *AmpR*, *lac promoter*, *IRES*, et cetera) across other labs.

An example of a consequential “smoking gun” is the *pAAV-CAG-tdTomato (codon diversified)* plasmid from Edward Boyden’s lab (Fig. 4c). A region of this plasmid corresponds to a pair of restriction sites, *KpnI*/*BamHI* (3588–3599), adjacent to one of the plasmid inserts. Disrupting only 12 bp of this sequence makes the plasmid appear as though it is from Roger Tsien’s lab. Note that the origin is also associated with the Boyden lab and changing it causes the plasmid to look as though it came from the Liquin Luo lab.

Most plasmids do not have a single “smoking gun” but rather have a combination of signals that collectively contribute to the prediction. The *pT7CFE1-TelR15-YPet* plasmid from Thoru Pederson’s lab is a good example (Fig. 4d). The lab-of-origin is correctly predicted by the particular combination of features that individually are prevalent across the Addgene dataset. If regions of the *T7 promoter*, yellow fluorescent protein (*YPet*), ampicillin resistance gene (*AmpR*), or the f1 origin of replication (*f1 ori*) are obscured, then the plasmid looks like it is from Christopher Voigt’s lab. Obscuring regions of the *IRES*, *YPet*, *AmpR*, or *f1 ori* makes it look like it is from Susan Lindquist’s lab. Other intergenic regions, when obscured, can make the plasmid appear to come from six different labs. Thus it is the collective impact of all these design choices that lead to a prediction, analogous to correctly identifying a noun by playing “20 questions.”

## Discussion

This work demonstrates that engineering DNA leaves design signatures that can be extracted from public repositories and used to guide an investigation into attribution. Over time, this will become more powerful for two reasons. First, designs in synthetic biology are growing in complexity and require more parts, genes, and genome modifications, thus leading to more “hooks” to create a unique signature^59–61^. Design tools are causing engineered and natural sequences to further diverge and thus easier to distinguish. Second, DNA datasets for training are growing and becoming more comprehensive, for example, Genbank doubles every 18 months and other databases are growing rapidly^62,63^. DNA synthesis companies, academic and industrial Bio-Foundries, and experimental cloud labs also act as centralized nodes of sequence information^64–69,76^.

Tracing a DNA sequence to a perpetrator requires a combination of investigative tools, some traditional and some high-tech^4^. To this end, artificial intelligence will be a powerful tool for microbial forensics, which is currently poorly equipped to assess engineered biology. It is important to note that detecting lab-specific signatures within a DNA sequence does not, in itself, indicate that that it is from that lab. Making an analogy with the Unabomber, determining a battery is from Duracell obviously does not mean that a Duracell employee is the perpetrator. Similarly, the tracing of the *Samonella* strain used by Rajneeshee to the commercial supplier ATCC (Rockville, MD) does not implicate ATCC but rather provides investigable clues around those that accessed or ordered it (an invoice was found at the cult compound) and points to where they may have been trained. The ability to scan engineered DNA sequences for signatures associated with individuals, labs, centers, and even countries provides actionable information to guide an investigation. Different regions of the DNA may be associated with different origins and this tool helps parse these hierarchal structures, similar to looking for shared code across computer viruses. Looking into the future, counters to this technology could also be developed, including sophisticated machine learning approaches, such as variational autoencoders^70^ and generative adversarial networks^71^, that can learn how to “fool” a classifier algorithm. These approaches represent a more sophisticated version of classical approaches to avoid detection, for example, altering sequence to subvert a PCR diagnostic^72^. Our approach could extent to applications beyond investigating sequences of malicious intent, for example, determining that a sequence from the environment is engineered and tracing its origins. The ability to rapidly parse DNA sequence information to guide the process of attribution and the understanding of potential countermeasures are capabilities critical to a national biodefense strategy. This work is an early step in achieving this, which will ultimately require more powerful algorithms, large sequence databases, and a concerted effort to address needs for forensics and attribution^73^.

## Methods

### Plasmid dataset

Plasmid DNA sequences and metadata were obtained in the form of a JavaScript Object Notation (JSON) file obtained upon request from Addgene, dated February 2016. We parsed this file to record each plasmid’s associated PubMed ID (PMID) and Digital Object Identifier (DOI). To determine a plasmid’s publication year, we first attempted to locate the PMID within a locally stored manuscript ID conversion file (ftp://ftp.ncbi.nlm.nih.gov/pub/pmc/PMC-ids.csv.gz). If the PMID was not found, we then attempted to locate the corresponding DOI. If either the PMID or DOI was found, then the publication year was stored. If neither could be found within the locally stored file, then a PubMed lookup tool (https://pypi.python.org/pypi/pubmed-lookup) was used to request the information from the NCBI server. While slower than the local approach, we were able to locate all remaining publication dates with the exception of 15 plasmids, which had no date information provided and were excluded from Fig. 1b. We also parsed the JSON file to store each plasmid’s depositing lab and associated DNA sequences. For plasmids with multiple depositing labs listed, the lab combination was treated as its own unique depositor. All unique depositors were then ranked based on their number of deposited plasmids (Fig. 1c). Plasmid DNA sequences in the JSON file came labeled as either: (1) “Full Repository”, where the entire DNA sequence was submitted by Addgene, (2) “Full Depositor”, where the depositing lab submitted the entire DNA sequence, (3) “Partial Repository”, where one or more sections of the plasmid were submitted by Addgene, or (4) “Partial Depositor”, where one or more sections of the plasmid were submitted by the depositing lab. We summed the total number of nts associated with a plasmid in each category (and also the sum of all four categories) and then ranked the plasmids accordingly (Fig. 1d).

### Input pre-processing and encoding

In order to have sufficient plasmid sequences to learn lab-of-origin from, we first eliminated any labs and their associated plasmids if the lab had deposited fewer than nine plasmids. If a plasmid had associated DNA sequence that came categorized as Full Repository, we used only that DNA sequence for training and ignored all other associated sequence information. If there was no Full Repository DNA sequence, but instead there was Full Depositor sequence, we used only that DNA sequence for training and ignored other sequences. If instead there was only Partial Repository and/or Partial Depositor DNA sequence for a plasmid (often resulting from Sanger sequencing reads), we concatenated all such partial sequences separated by spacer sequences of 48 Ns to create the training sequence.

Subsequently, to reduce training time we truncated long DNA sequences to 8000 nts. In the rare case that any DNA sequence characters were not A, T, G, C, or N, the character was converted to N. We padded the resulting sequences with Ns to a total length of 8000 bp, and then concatenated the sequence’s reverse complement to itself separated by a spacer sequence of 48 Ns. Lastly, we encoded each nt in the final sequence as a one-hot vector where A = [1 0 0 0], T = [0 1 0 0], G = [0 0 1 0], C = [0 0 0 1], and N = [0 0 0 0] (Fig. 2a). Similarly, the identity of the depositing lab was also encoded as a one-hot vector with length 827. These one-hot vector sequence inputs and depositor labels were used to train the neural network.

### Convolutional neural network

We implemented and trained neural network architectures using Keras (version 2.0.4) using the Tensorflow backend (version 1.1.0) in Python (version 3.5.2) with NumPy (version 1.13.0) and SciPy (version 0.19.0). Other packages include Pickle for data serialization, json (version 2.0.9) to parse the Addgene data file, and pubmed_lookup (version 0.2.1). Neural networks were trained on an NVIDIA GRID K520 GPU using Amazon Web Services Elastic Cloud Compute (EC2) running Ubuntu 16.04.1 LTS.

The CNN architecture comprises from input to output: the 16,048 × 4 one-hot vector DNA sequence input layer, a single convolutional layer with multiple filters, a max-pooling layer for each filter over the entire input length, a batch normalization layer, a fully connected layer, a second batch normalization layer, a second fully connected layer where each node corresponds to a depositing lab, and a conversion to probability using the softmax function. Softmax is computed by taking the signal *z*_*j*_ generated for each lab’s output node *j* and converting to probability using the equation: $\sigma \left( {z_j} \right) = \frac{{e^{z_j}}}{{\mathop {\sum }\nolimits_k e^{z_k}}}$.

For the convolutional layer, we used the Keras border mode *same* to generate output vectors with the same size as the input. Batch normalization layers (used after the max-pool layer and the first fully connected layer) have been shown to accelerate deep network training by reducing covariate shift^74^. We used the rectified linear unit (ReLU) activation function for the convolutional and fully connected layers, the *adam* optimizer function for training, a categorical cross-entropy loss function to back-propagate errors from the output, and a mini-batch size of 8 inputs per update. To compensate for skewed plasmid deposit numbers across labs, we used the Keras *class_weight* variable to weight the loss function during training by the reciprocal of training plasmid number for that lab.

Training data was split into six subsets due to memory constraints, and subsets were loaded and trained sequentially during each epoch. After training for 100 epochs, we stored the final neural network architecture and parameters for downstream use. We visualized filter weights (Fig. 2a) by converting the per-position nt filter weights (*w*) to Boltzmann factors using the following formula, where the temperature parameter *T* was selected by eye to maximize contrast in a heatmap: $f\left( w \right) = e^{ - w/T}$

To calculate the number of DNA sequences that could be analyzed per second, we used the validation and cross-validation sets each containing 2481 plasmid sequences. Before timing, we pre-processed the input data, concatenated the sequence with 48 Ns followed by the reverse complement sequence, and then encoded them as one-hot vectors (previous section). Using the stored neural network architecture from above, we predicted lab-of-origin for the entire encoded validation and cross-validation sets while tracking the seconds elapsed. Evaluation took 40.5 s to make predictions for both sets, which corresponds to 4962 sequences of 8000 bp each, or a rate of 980,000 bp s^−1^.

### Bayesian optimization of hyperparameters

To explore different hyperparameter sets for filter length, number of filters, and number of fully connected nodes, we used a Bayesian optimization technique that models the generalization performance of the hyperparameter landscape as a Gaussian process^75^. We used a Bayesian optimization Python package available on Github (https://github.com/fmfn/BayesianOptimization).

We optimized for validation accuracy per wall-clock time at the end of 5 epochs using the “upper confidence bound” exploration strategy with *alpha* equal to 10^−5^ and *kappa* equal to 5. We varied the number of convolutional filters between 1 and 512, the length of the convolution filters between 1 and 48 nts, and the number of nodes in the fully connected layer between 1 and 256. Hyperparameter upper bounds were chosen due to memory constraints. Training 23 different architectures yielded a range of training accuracies, validation accuracies, and wall-clock times. Many architectures failed to learn after five epochs, and these were characterized by few filters, small filter length, or both. The hyperparameters resulting in the greatest validation accuracy per training time (128 filters, a filter length of 12 nt, and 64 fully connected nodes) were selected for extended training.

### Permutation of plasmid authorship

As an additional control to test for overfitting, we scrambled the depositing lab labels across all the plasmids and then split them into training, validation, and cross-validation sets. Plasmid DNA sequences were unmodified. The frequency of each lab from the non-scrambled dataset was maintained in the scrambled dataset and we used the *class_weight* variable in the same manner as above to compensate for differences in lab depositing frequency. We trained the network for 100 epochs, after which the validation accuracy was 0.04%, comparable to what would be expected from randomly choosing a lab from the list (0.12%).

### Simulation and calculation of *P* values

To determine the statistical significance of pre-softmax neuron activities generated by the CNN, we calculated *P* values from an activity distribution for random DNA sequences^51,52^. We first generated 10^4^ random DNA sequences of length nt with the same frequencies of A, T, G, and C as the Addgene training set. We repeated this for DNA sequence lengths 1000 nt, 3685 nt (the length of *pCI-YFP*), and 8000 nt (the maximum allowable length in our architecture). For each random DNA sequence, we padded and concatenated the sequence reverse complement in the same manner as the Addgene set (see above), before converting the sequence to a one-hot encoding. We then used the trained CNN to compute the maximum pre-softmax neural activity across all labs for each sequence. A normalized histogram of the max activities can be approximated by an extreme value distribution, which has a cumulative distribution function *of y* = exp(−exp(−*λ*(*x*−*μ*))). The probability of observing an activity, *A*, greater than *x* is therefore *P*(*A* > *x*) = 1−exp(−exp(−*λ*(*x*−*μ*))), where *λ* is the steepness parameter and *μ* is the distribution’s offset from 0. We fit the empirical distributions to this equation, which was then used to calculate the *P* value of the pre-softmax neural activity for a DNA sequence of length N.

### BLAST analysis

For the alignment of the *pCI-YFP* sequence to sequences in Genbank, BLAST was performed against the nr/nt nt collection using the web interface (https://blast.ncbi.nlm.nih.gov/Blast.cgi). Default parameters were used except the Entrez Query is “NOT genome,” Max target sequences was 20,000, Expect threshold was 0.1, and the Max matches in a query range was 5000. The match/mismatch scores are 1,−2, and the gap cost was linear. For plasmid features *KanR*, *p15A*, and *YFP*, web BLAST was performed against the nr/nt collection using the megablast default parameters and the number of alignments reported. Additionally, for each feature BLAST was performed locally against the training set and the number of alignments in the output file recorded. To do so, FASTA files were created for each feature, and a BLAST database was created from a FASTA file with all Addgene training DNA sequences. For a FASTA file containing the entire *pCI-YFP* sequence, BLAST was performed against the Addgene training set and the scores for the top 10 labs were recorded (Fig. 3b). The alignment of the entire *pCI-YFP* plasmid to the Addgene training set returned >10^4^ matches. We found the smallest non-zero *E*-value (4 × 10^−180^) and then counted the number of alignments with an *E*-value of zero to conclude that 11,369 plasmid alignments have an *E*-value < 10^−180^.

### Mutational trajectories

Point mutations were introduced to the *pCI-YFP* DNA sequence iteratively over 1000 generations for a single trajectory. Each point mutation in a trajectory was made at a unique position compared to all previous mutations (i.e., mutation positions were selected without replacement). The identity of the nt post-mutation was one of the three nts not present at that position pre-mutation. After each mutation, the new DNA sequence was evaluated by the fully trained neural network and the probability prediction for Christopher Voigt’s lab was recorded. Thirty such mutational trajectories were performed, each starting from the original *pCI-YFP* sequence. The geometric mean of the probabilities at each mutation step was calculated.

### Sequence scanning to determine signature

In order to determine the importance of different regions within a plasmid toward the predictions, a window of 50 Ns was scanned across the plasmid to mask the underlying sequence at each position. For each plasmid, the starting DNA sequence was the training DNA sequence before it was padded to a length of 8000 bp and concatenated with its reverse complement. Using this sequence, a periodic boundary condition was applied so that, when any of the Ns in the 50 N scanning window extended past the end boundary of the DNA sequence, those Ns were placed back at the beginning of the DNA sequence. For each position of the window scan, the resulting masked DNA sequence was padded to 8000 bp, concatenated with a spacer of 48 Ns followed by the reverse complement, converted to a one-hot vector, and input into the fully trained neural network. The pre-softmax neural activities and softmax probabilities for the top predicted labs were evaluated. For each nt in a plasmid, the CNN predictions from all 50 frames where the sliding window masked that nt were averaged and visualized (Fig. 4, line traces). The top lab prediction for each position of the masking window was also visualized (Fig. 4, colored bars, widths not to scale).

### Code availability

Source code is available from Github at https://github.com/VoigtLab/predict-lab-origin.

### Data availability

The authors declare that all data supporting this study are available within the article and its Supplementary Information file or are available from the corresponding author upon request.

## Electronic supplementary material

Supplementary Information
