# Supplementary material for: Deep learning to predict the lab-of-origin of engineered DNA
Source: Nat Commun. 2018 Aug 7;9:3135. doi: 10.1038/s41467-018-05378-z (PMC6081423; doi:10.1038/s41467-018-05378-z)
Supplement: Supplementary file 1 — Supplementary Information [file 41467_2018_5378_MOESM1_ESM.pdf]

Supplemental Information for:

Deep learning to predict the lab-of-origin of engineered DNA

Nielsen et. al

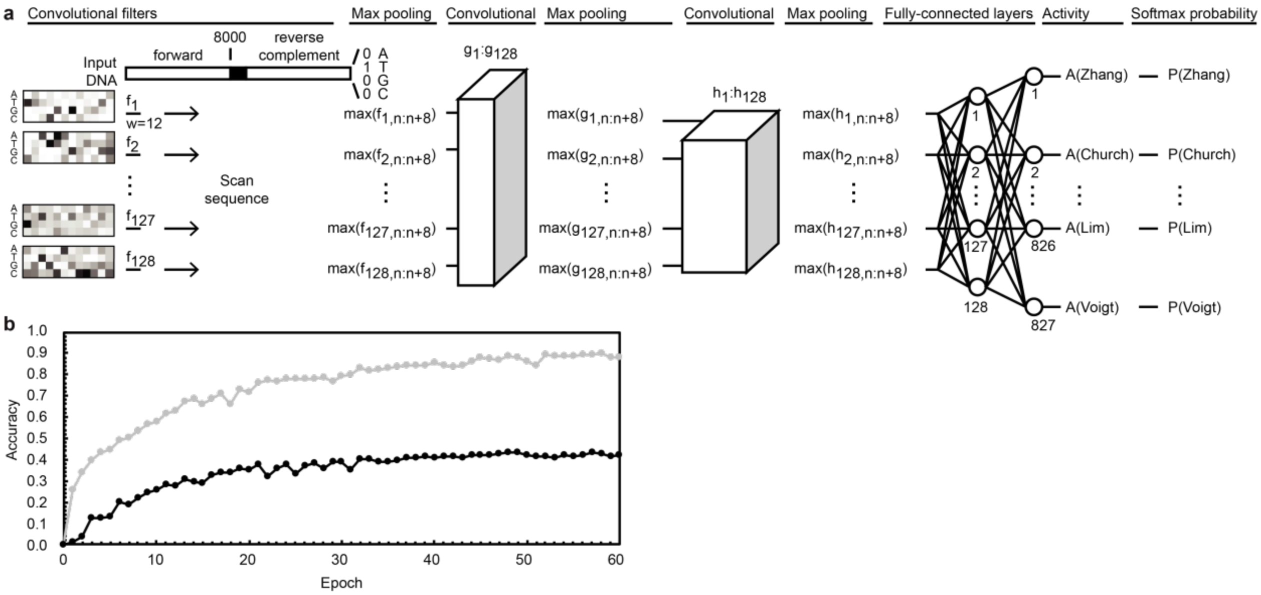

Supplementary Figure 1: ***Hierarchical convolutional neural network accuracy. (A)*** *Hierarchical convolutional neural network architecture. DNA sequences are converted to 16,048 x 4 matrices, where the identity of each nucleotide is converted to a one-hot vector. This input feeds into three convolutional layers, each with 128 filters, a filter length of 12, and a downstream max-pooling layer over 8 positions. The activations feed into two fully connected layers, which generates neural activity predictions for each lab, A(Name), before behind converted to probabilities using the softmax function, P(Name). The lab prediction is taken as the highest softmax probability output. Batch normalization layers are not shown.* ***(B)*** *Training accuracy (gray) and validation accuracy (black) per epoch for the chosen architecture.*

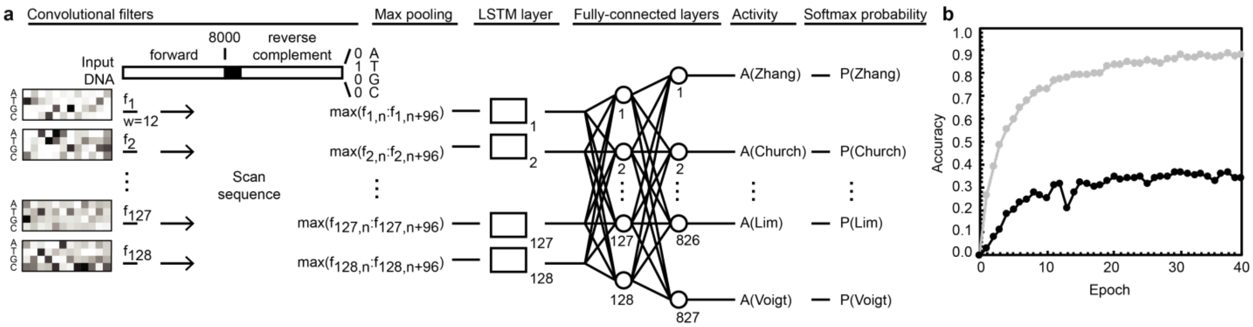

Supplementary Figure 2: ***Recurrent neural network accuracy.*** ***(A)*** *Recurrent neural network architecture. DNA sequences are converted to 16,048 x 4 matrices, where the identity of each nucleotide is converted to a one-hot vector. This input is scanned by 128 convolutional filters (f_1 to f_{128}) each with a width, w, of 12 nucleotide positions. The maximum activation for each filter in each window of 96 nucleotides, max($f_{1,n}$:$f_{1,n+96}$), across the entire input sequence is taken. Each sequence of max-pool activations are fed into one of 128 LSTM units, which then feed into two fully connected layers, which generates neural activity predictions for each lab, A(Name), before behind converted to probabilities using the softmax function, P(Name). The lab prediction is taken as the highest probability output. Batch normalization layers are not shown.* ***(B)*** *Training accuracy (gray) and validation accuracy (black) per epoch for the chosen architecture.*

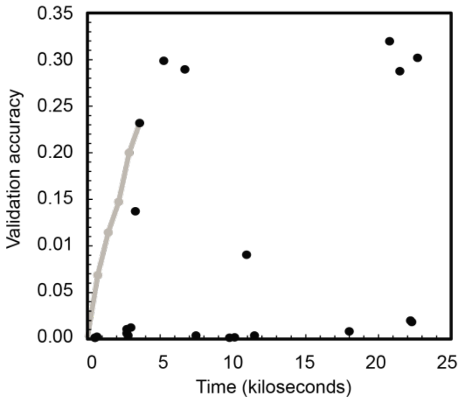

Supplementary Figure 3: ***Convolutional neural network hyperparameter optimization.*** *Validation accuracy versus time elapsed after 5 epochs shown for 23 hyperparameter sets (black points). The architecture with the highest validation accuracy per time was chosen, and the trajectory from epoch 0 to 5 is shown (gray line).*

Supplementary Note 1: Alternative architectures and hyperparameter optimization

1.A. Hierarchical convolutional neural network architecture

Neural networks with many convolutional layers have been widely used for computer vision applications[1,2]. Each layer in the progression learns increasingly abstract and large-scale features in the input data. We reasoned that hierarchical architectures could effectively learn short, local DNA motifs at the first layer, and higher-order spatial relationships in downstream layers.

We tested a network where the one-hot encoding, fully-connected layers, softmax probability, and training parameters were the same as Figure 2. However, a series of three convolutional layers is used in place of the single convolutional layer (Supplementary Figure 1). Each of these convolutional layers has 128 filters, a filter length of 12, and is followed by a max-pooling layer. Each max-pooling layer outputs the maximum upstream convolutional filter activation over a sliding window of 8 positions, rather than the entire input sequence. A batch normalization layer is used after each max-pooling layer and after the first fully-connected layer.

With this architecture, training accuracy achieves 90% over 60 epochs and the validation accuracy reaches 43%. While hierarchical architectures had slightly lower validation accuracy than the single convolutional layer architecture in Figure 2, they took twice as long to train so were not selected for further study.

1.B Recurrent neural network architecture

Recurrent neural networks have been successfully applied to non-coding DNA sequence classification by Quang and Xie[1] . We explored a similar neural network design by inserting Long Short-Term Memory (LSTM) units[2] between the convolutional and fully connected layers (Supplementary Figure 2). LSTM units take sequential data as input; at each step in a sequence, an LSTM unit accepts input data and also the LSTM unit's previous state. Over the course of training through backpropagation, LSTM units can learn what features to remember in order to minimize training loss.

In our network, the one-hot encoding, convolutional layer, fully-connected layers, softmax probability, and training parameters are identical to Figure 2. However, the max-pooling layer is modified so that it outputs the maximum convolutional filter activation over a sliding window of 96 nucleotides, rather than the entire input sequence. A batch normalization layer is used after the convolutional layer and the first fully-connected layer. Convolutional filters each generate a sequence of 668 max-pool signals across the entire input sequence. Each of these sequences feeds into one of 128 LSTM units, which then feed into the fully-connected layer of 128 nodes. Training accuracy achieves 89% over 40 epochs, however the validation accuracy plateaus at 36%, which is indicative of overfitting. Convolutional neural networks have recently been shown to outperform recurrent neural networks across an array of tasks[3] , and our results are consistent with this observation.

1.C Bayesian optimization

Once the architecture for the convolutional neural network was chosen, we used Bayesian optimization to explore hyperparameters for filter length, number of filters, and number of fully-connected nodes (Methods). This technique models hyperparameter generalization performance as a Gaussian process[6]. We optimized for validation accuracy per wall-clock time at the end of 5 epochs. The maximum validation accuracy per training time was achieved with 128 filters, a filter length of 12 nt, and 64 fully-connected nodes (Supplementary Figure 3).

Supplementary References

1. Krizhevsky, A., Sutskever, I. & Hinton, G. E. ImageNet Classification with Deep Convolutional Neural Networks. in *Advances in Neural Information Processing Systems 25* (eds. Pereira, F., Burges, C. J. C., Bottou, L. & Weinberger, K. Q.) 1097–1105 (Curran Associates, Inc., 2012).

2. Simonyan, K. & Zisserman, A. Very Deep Convolutional Networks for Large-Scale Image Recognition. *ArXiv14091556 Cs* (2014).

3. Quang, D. & Xie, X. DanQ: a hybrid convolutional and recurrent deep neural network for quantifying the function of DNA sequences. *Nucleic Acids Res.* **44,** e107–e107 (2016).

4. Hochreiter, S. & Schmidhuber, J. Long Short-Term Memory. *Neural Comput.* **9,** 1735–1780 (1997).

5. Bai, S., Kolter, J. Z. & Koltun, V. An Empirical Evaluation of Generic Convolutional and Recurrent Networks for Sequence Modeling. *ArXiv180301271 Cs* (2018).

6. Snoek, J., Larochelle, H. & Adams, R. P. Practical Bayesian Optimization of Machine Learning Algorithms. in *Advances in Neural Information Processing Systems 25* (eds. Pereira, F., Burges, C. J. C., Bottou, L. & Weinberger, K. Q.) 2951–2959 (Curran Associates, Inc., 2012).
